# Editorial: Evidence-based outreach/service-learning to improve health-related self-efficacy through STEMM education

**DOI:** 10.3389/fpubh.2025.1678120

**Published:** 2025-09-09

**Authors:** Kirsten A. Porter-Stransky, Carolina Restini, Peter J. Vollbrecht, Tracey Weiler, Jonathan J. Wisco

**Affiliations:** ^1^Department of Biomedical Sciences, University of South Carolina School of Medicine Greenville, Greenville, SC, United States; ^2^Department of Pharmacology and Toxicology, College of Osteopathic Medicine, Michigan State University (Macomb University College-MUC, and Detroit Medical Center-DMC), Clinton Township/Detroit, MI, United States; ^3^Department of Biomedical Sciences, Western Michigan University Homer Stryker M.D. School of Medicine, Kalamazoo, MI, United States; ^4^Department of Medical Education, Herbert Wertheim College of Medicine, Florida International University, Miami, FL, United States; ^5^Department of Anatomy and Neurobiology, Boston University Aram V. Chobanian & Edward Avedisian School of Medicine, Boston, MA, United States

**Keywords:** community engagement, outreach, service-learning, health self-efficacy, public health, empowerment

Health professions institutions, including medical schools, have long been involved in outreach and service learning to simultaneously serve the local community and provide hands-on educational opportunities for health professions students (1, 2). Many such programs seek to enhance public health, foster an interest in science for K-12 students, or provide pathways to health professions schools for individuals traditionally underrepresented in science and medicine. The need for such programs remains, especially with continued health disparities evident in rural and low socioeconomic status communities (3), the lack of parity in the physician workforce (4), and growing skepticism of science and public health since the COVID-19 pandemic (5).

There is a critical need to apply a scholarly approach to the development, implementation, and programmatic assessment of outreach and service-learning programs, followed by intentional dissemination of these programs and their outcomes so that the field can generate evidence-based best practices in outreach and service-learning. To help fill this gap, we created the Research Topic “*Evidence-based outreach/service-learning to improve health-related self-efficacy through STEMM education*” in *Frontiers in Public Health*. As Guest Editors for this Research Topic, we are delighted to present the collection of innovative articles comprising this Research Topic. This Research Topic unites diverse research that leverages science, technology, engineering, math, and medicine (STEMM) education and community engagement to empower both individuals and communities while enhancing public health. This editorial summarizes the papers within the Research Topic, exploring four key themes, each highlighting a critical aspect of how evidence-based outreach and service-learning programs enhance health literacy, promote equity, and inspire the next generation of health professionals (Figure 1).

**Figure 1:**
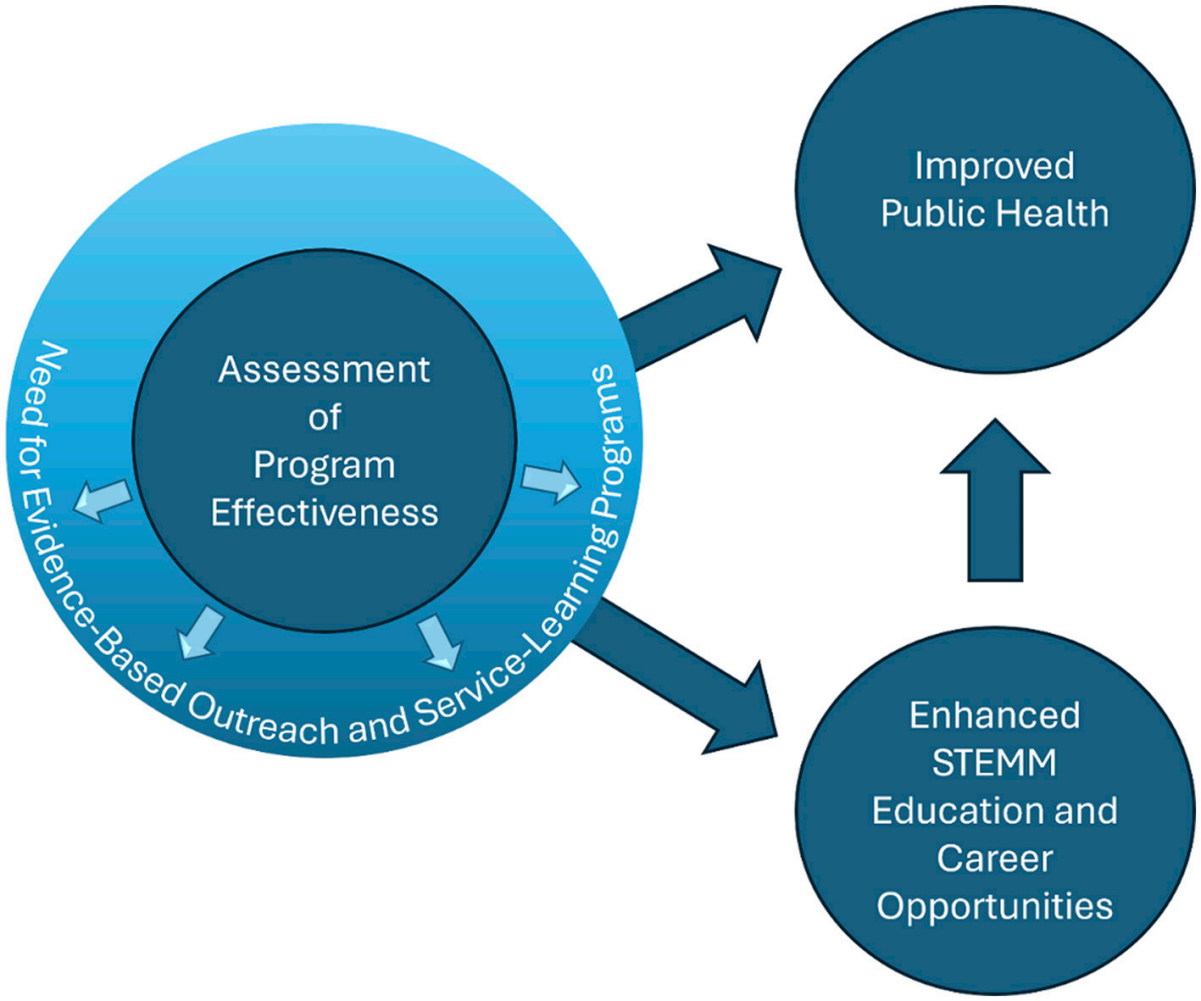
Relationships among themes represented in research collection. Evidence-based outreach and service-learning programs benefit from constant and consistent evaluation and assessment. Effective programs can lead to enhanced STEMM education and career opportunities for individuals, while simultaneously contributing to the improvement of public health in communities as a function of individuals collectively improving their health self-efficacy. Individuals that enter STEMM healthcare careers can also improve public health awareness and practices in the communities of their service and work.

## Theme 1: The need for evidence-based outreach and service-learning programs

Two studies lay the foundation by underscoring the importance of structured, evidence-based outreach and service-learning initiatives. (1) Restini et al. present a framework for developing effective programs that resonate with diverse populations. “*Empowering the future: improving community wellbeing and health literacy through outreach and service-learning*” demonstrates how targeted programs foster health-related self-efficacy, equipping individuals and communities with the knowledge and confidence to manage their health. (2) Porter-Stransky et al. investigate the relationship between socioeconomic factors and perceptions of science, emphasizing the need for inclusive outreach to bridge gaps in STEMM engagement.

## Theme 2: Outreach programs to improve public health

A substantial portion of this Research Topic focuses on outreach programs addressing important public health challenges. Several studies showcase innovative community-based interventions initiated during the COVID-19 pandemic. For instance, Ban et al. and Bekele et al. illustrate how tailored approaches improved vaccine uptake among underserved groups in Missouri and Atlanta, respectively. Similarly, Johnson et al. and Pichon et al. demonstrate the power of outreach paired with technology to improve medical access for vulnerable populations. Other papers, such as Kai et al. and Holcomb et al. highlight scalable models for health and cancer screening. Finally, Belkora et al. demonstrate how service learning strengthens public health infrastructure, providing a model for workforce development.

## Theme 3: Programs to enhance STEMM education and career opportunities

This theme combines evidence of programs that explore health and science education to empower individuals to pursue STEMM career paths, ranging from elementary school to medical school. Butterfield et al. taught elementary school children basic anatomy, physiology, and nutrition, fostering an early interest in health and STEMM. Imeh-Nathaniel et al. educated middle schoolers on stroke risk factors and interventions. Similarly, Mytting et al. showcased healthcare career options to inspire rural youth to become health professionals serving rural British Columbia. Anders et al. developed a summer program for students underrepresented in STEMM in a rural, low-income state to increase biomedical research career interest and opportunity. Finally, Salas et al. developed an innovative pilot program whereby preclinical students develop STEMM outreach curricula, enhancing their learning while serving the community.

## Theme 4: Effectiveness of health and science outreach, service-learning, and pathway programs

This last theme focuses on the importance of evaluating the impact and effectiveness of such programs. Vollbrecht et al. demonstrated multiple methods to assess whether outreach and service-learning programs are meeting their stated goals. Affini et al. examined demographic characteristics of participants in a health profession recruitment and exposure program in Chicago. Naik et al. and Kemp et al. used quantitative methods to parse the relationships between educational experiences and strategies that improve college readiness and self-efficacy for STEMM. Ha et al. used qualitative methods to assess parents' views on the acceptability of a physical literacy program, and van Dongen et al. used mixed methods to investigate the impact of an intervention to enhance physical activity and a healthy diet in secondary schools. Finally, Prabhakar et al. investigated medical student engagement during teaching science through community-based outreach programs, with the goal of improving communication skills and engagement.

## Conclusion

This Research Topic illustrates the transformative potential of evidence-based outreach and service-learning in improving career opportunities and public health through engagement in STEMM across the educational continuum. By addressing health literacy, equity, and career development, these studies offer actionable insights for educators, public health practitioners, and policymakers. The findings highlight the importance of culturally tailored, data-driven and evidence-based programs that empower communities both through direct intervention and by inspiring future healthcare professionals. As we move forward, these models can guide efforts to build healthier, more equitable societies through STEMM education.

We thank the authors for their contributions and the *Frontiers in Public Health* editorial team for their support of this Research Topic. We hope this Research Topic inspires continued innovation in public health outreach and education.
